# Effect of Current-Season-Only Versus Continuous Two-Season Influenza Vaccination on Mortality in Older Adults: A Propensity-Score-Matched Retrospective Cohort Study

**DOI:** 10.3390/vaccines13020164

**Published:** 2025-02-08

**Authors:** Huimin Sun, Yijing Wang, Yongyue Wei, Weihua Hu, Junwen Zhou, Nuosu Nama, Yujie Ma, Gang Liu, Yuantao Hao

**Affiliations:** 1 Department of Epidemiology and Health Statistics, School of Public Health, Peking University, Beijing 100191, China; kellysun@pku.edu.cn (H.S.); ywei@pku.edu.cn (Y.W.); huwh5@bjmu.edu.cn (W.H.); 1810306204@pku.edu.cn (J.Z.); nuosunama@bjmu.edu.cn (N.N.); simone@bjmu.edu.cn (Y.M.); 2 Shenzhen Center for Disease Control and Prevention, Shenzhen 518055, China; wangyj3@mail2.sysu.edu.cn; 3 Peking University Center for Public Health and Epidemic Preparedness & Response, Beijing 100191, China; 4 Key Laboratory of Epidemiology of Major Diseases (Peking University), Ministry of Education, Beijing 100191, China

**Keywords:** influenza vaccination, older adults, mortality, cardio-cerebral vascular diseases, survival analysis

## Abstract

Background/Objectives: This study evaluated the impact of influenza vaccination on mortality using real-world data and compared the effect of current-season-only vaccination versus continuous two-season vaccination. Methods: The 2017–2019 data from the Center for Disease Control and Prevention of Shenzhen, Guangdong, China, included 880,119 individuals aged ≥65 years. The participants were divided into vaccinated and unvaccinated groups and matched using propensity scores with a 1:4 nearest-neighbor approach. Vaccinated individuals were further divided into current-season-only and continuous two-season vaccination groups, matched 1:1. Cox’s multivariable proportional hazards regression models were used to assess the effect of vaccination on all-cause mortality, with Firth’s penalized likelihood method applied to correct for a few events. The Fine–Gray competing risk models were used to assess the effect of vaccination on cardio-cerebral vascular disease (CCVD) mortality. Sensitivity analyses, including caliper matching, a nested case–control design, and Poisson’s regression, were performed to test the robustness of the results. Results: Influenza vaccination reduced all-cause mortality by 39% (HR = 0.61, 95% CI: 0.47–0.80) and 55% (HR = 0.45, 95% CI: 0.33–0.60) in 2017–2018 and 2018–2019, respectively. Current-season-only vaccination showed stronger protective effects than continuous two-season vaccination (HR = 0.56, 95% CI: 0.31–0.99). Influenza vaccination reduced CCVD mortality by 46% (HR = 0.54, 95% CI: 0.34–0.84) in 2018–2019. The results were consistent across the sensitivity analyses. Conclusions: Influenza vaccination was associated with a reduced risk of all-cause and CCVD mortality in older adults, underscoring the importance of routine influenza vaccination in older populations. Stronger effects were observed for current-season-only vaccination, warranting further research to confirm the association and explore mechanisms.

## 1. Introduction

Seasonal influenza can cause significant morbidity and mortality in all age groups, with an estimated 290,000–650,000 respiratory deaths per year worldwide [1]. Older adults are more vulnerable due to declining physical function and compromised pulmonary immune defenses [2], and previous studies have shown that 80–95% of excess influenza-associated mortality occurs in older adults ≥ 65 years of age [3]. The World Health Organization (WHO) suggests that everyone six months of age and older be vaccinated against influenza, with older adults as a priority group [4]. Due to antigenic drift, influenza viruses mutate frequently, making it necessary to reformulate and revaccinate influenza vaccines annually. Typically, protective antibodies are produced two to four weeks after influenza vaccination, and protection can be maintained for up to six months [5]. A meta-analysis by Young et al. showed that in older adults aged ≥65 years, antibody titers were close to pre-vaccination levels after one year [6].

There has been much research supporting the significant protective effect of influenza vaccination against mortality [7,8,9,10,11]. However, vaccine effectiveness may decrease with repeated doses due to weakened vaccine-induced immunity to homologous strains. Evidence from a meta-analysis in 2023 showed a 7–18% reduction in the protective effect of influenza vaccination for two continuous seasons compared with the current season alone [12]. The review by Belongia et al. indicated that the negative effects of repeated vaccination were most pronounced for the A H3N2 subtype [13]. The meta-analysis by Okoli et al. of publications from 2011 to 2020 suggested that the influenza vaccine effectiveness (VE) declined as the maturity of influenza vaccination programs increased, with a more significant decline observed in individuals aged ≥65 years [14]. The meta-analysis of randomized controlled trials by Keitel et al. found that participants who received influenza vaccination for two continuous seasons had a considerably lower likelihood of seroconversion [odds ratio (OR) = 0.76, 95% CI: 0.60–0.96], while those vaccinated in the current season showed an 18% higher geometric mean antibody titer (OR = 1.18, 95% CI: 1.04–1.33) [15]. Yet, epidemiologic studies to date have almost exclusively compared current-season-only and continuous two-season vaccination with an unvaccinated group and then evaluated the difference in effectiveness between the two. The potential challenge with this approach is using the unvaccinated population as the reference group, which only allows for estimation of the effect of each vaccination strategy relative to no vaccination. Even calculating the difference in VE between continuous vaccination and current-season-only vaccination provides limited insight into their true differences in protective effectiveness. To date, few studies have directly compared the effects of these two vaccination strategies. Given the increasing attention and concern among researchers regarding whether continuous influenza vaccination is associated with reduced protective effectiveness, it is imperative that direct comparisons be conducted to investigate whether continuous vaccination leads to a decline in protective effectiveness compared to current-season-only vaccination and to quantify the extent of any reduction.

The WHO and the Council of the European Union have recommended that countries set a target influenza vaccination coverage rate of 75% for priority groups [16,17]. However, a nationwide cross-sectional study reported that the influenza vaccination coverage rate among older adults aged ≥60 years in China was only 3.8% [18]. In comparison, the coverage rate among older adults aged ≥60 years in Germany was 47.3% during the 2020–2021 season [19], while in the United States, the influenza vaccination coverage rate among older adults ranged from 69.6% to 75.0% between the 2008–2009 and 2017–2018 seasons [20]. Similarly, in Japan, influenza vaccination coverage among older adults consistently exceeded 40% between 2007 and 2017 [21]. These findings highlight the persistently low influenza vaccination coverage among older adults in China. In recent years, some regions in China have begun to implement a policy of free influenza vaccination for certain groups of people with financial support from the government. For example, the Shenzhen government has included free influenza vaccination for people aged 60 years and older as a livelihood benefit since 2016, with the vaccination taking place from each October to the following April. Trivalent inactivated influenza vaccine (IIV3) is primarily used, followed by quadrivalent inactivated influenza vaccine (IIV4), and the vaccine strain formulations are consistent with the components published annually by the WHO. The aim of this study was to evaluate the effect of influenza vaccination on mortality in older adults based on real-world data from Shenzhen, China, from 2017 to 2019, and to compare the difference in effect between current-season-only vaccination and continuous two-season vaccination so as to provide a scientific basis for influenza vaccination strategies and health care for older adults.

## 2. Materials and Methods

### 2.1. Data Sources

Shenzhen is located in the subtropics, where the seasonal pattern of influenza is more complex, with peaks of influenza epidemics occurring in both winter and summer [22,23]. In this study, the 36th week of each year to the 35th week of the following year was divided into one influenza season, and the influenza vaccination records, demographic information, physical examination records, and mortality records of the older adults in Shenzhen for a total of two influenza seasons, from the 36th week of 2017 to the 35th week of 2019, were provided and anonymized by the Shenzhen Center for Disease Control and Prevention. The de-identified dataset contained the following variables: (1) influenza vaccination records: date of influenza vaccination, immunization program, vaccine type, and vaccine manufacturer; (2) demographic information: date of birth, gender, ethnicity, marital status, occupational type, education level, and insurance type; (3) physical examination records: diabetes, hypertension, body mass index (BMI), alcohol consumption, dietary habits, frequency of exercise, smoking, hemoglobin (HGB), and serum creatinine (SCR); and (4) mortality records: underlying cause of death, the International Statistical Classification of Diseases and Related Health Problems 10th Revision (ICD-10) code, and date of death.

### 2.2. Study Population

The dataset for this study was matched from three different sources, including the physical examination dataset, the influenza vaccination record dataset, and the mortality dataset, in which the mortality dataset could only obtain information on older adults aged 65 years and older. Thus, the study subjects were older adults aged 65 years and above in Shenzhen.

• Inclusion criteria: older adults who survived and were at least 65 years old at the start of the 36th week in 2017 and 2018 were included in this study;
• Exclusion criteria: (1) for individuals who died during the 2017–2018 and 2018–2019 influenza seasons without the date of death or the underlying cause of death, when the number was low (<1%), they were excluded, and (2) for individuals who received an influenza vaccine during the 2017–2018 and 2018–2019 influenza seasons lacking information on the vaccination date, when the number was low (<1%), they were excluded.

### 2.3. Determination of Control and Exposure Groups

In this study, retrospective cohort studies and survival analyses were conducted in the whole population and in the influenza-vaccinated population, respectively. Of the entire study population during the 2017–2018 and 2018–2019 influenza seasons, the control group was those who did not receive an influenza vaccine during each influenza season, and the exposure group was those who received an influenza vaccine during each influenza season. Among the study participants with documented influenza vaccination during the 2018–2019 influenza season, the control group was those who received an influenza vaccine during both the 2017–2018 and 2018–2019 seasons, and the exposure group was those who received an influenza vaccine only in 2018–2019 and were not vaccinated during the 2017–2018 influenza season.

Influenza vaccination was administered from the beginning of the annual influenza season (the 36th week) to two weeks before the end of the influenza season (the 33rd week of the following year).

### 2.4. Outcomes

The primary outcome was all-cause mortality, and the secondary outcome was mortality from cardio-cerebral vascular diseases (CCVDs). The observation period started from two weeks after vaccination for those vaccinated and from the 38th week of the year for those unvaccinated. Observation ceased by the end of the influenza season. According to ICD-10 codes, CCVD was defined using I00–I99.

### 2.5. Statistical Analyses

The raw data were reviewed to exclude illogical data, such as two records with ages exceeding the human life expectancy limit (120 years) [24]. Four variables in the dataset contained missing values, namely, ethnicity, education level, marital status, and occupational type, with percentages of missing values all less than three out of ten thousand, and the missing values were handled using multiple imputations [25]. Confounders between the exposure and control groups were balanced using propensity score matching in the form of nearest-neighbor matching without replacement. The matching ratio of the vaccinated group to the unvaccinated group was 1:4 in the entire population, and that of the current-season-only group to the continuous two-season group was 1:1 in the vaccinated population. Differences between groups were measured by the standardized mean difference (SMD) [26]. For all-cause mortality, Kaplan–Meier survival curves were plotted, and log-rank tests were performed. For CCVD mortality, cumulative incidence function curves were plotted, and Gray’s tests were performed. The impact of influenza vaccination on all-cause mortality was analyzed using Cox’s multifactorial regression with stepwise backward selection, selecting the model that minimized the Akaike Information Criterion to calculate hazard ratios (HRs) and 95% confidence intervals (CIs). To correct for immortal time bias caused by the misalignment between influenza season onset and vaccination timing, vaccination status was treated as a time-dependent variable based on the vaccination date in the Cox model. When examining the effect of current-season-only vaccination in the vaccinated population, Firth’s penalized partial likelihood method was applied to the Cox regression model to address the issue of a small number of death events [27,28]. Considering mortality from other causes as a competing event for CCVD mortality, the impact of influenza vaccination on CCVD mortality was analyzed using the Fine–Gray competing risks model.

To evaluate the robustness of the study under different propensity score matching methods, study designs, and statistical approaches, three sensitivity analyses were conducted: (1) Propensity score matching with caliper values was used, with the width of the caliper value equal to 0.2 of the standard deviation of the logit of the propensity score [29]. (2) Given the rarity of death events (<1%) in this study, a nested case–control design was adopted. Death cases were matched with surviving individuals at the time of death by age (±one year) and sex at a 1:10 ratio, followed by conditional logistic regression analysis. (3) As the occurrence of death events followed a Poisson distribution, Poisson’s regression was performed after propensity score matching.

To investigate whether age, gender, education level, insurance type, occupational type, BMI, HGB, hypertension, and diabetes had modifying effects on the association between influenza vaccination and all-cause mortality, this study conducted subgroup analyses in the total population with the above factors as subgrouping variables. The interaction terms were included in the model, and likelihood ratio tests were performed to determine whether the differences between strata were statistically significant. Due to the low number of deaths, subgroup analyses were not conducted in the influenza-vaccinated population. To explore the association between influenza vaccination and mortality from CCVD subtypes, we further categorized CCVD (ICD-10: I00–I99) into ischemic heart disease (ICD-10: I20–I25), cerebrovascular disease (ICD-10: I60–I69), and other circulatory system diseases (ICD-10: I00–I15, I26–I52, I70–I99). Competing risk models were applied to analyze the associations between influenza vaccination and these three specific mortality outcomes in the total population.

R 4.3.2 was used for data cleaning and analysis in this study. Statistical results were taken as a two-sided test with a level of α = 0.05.

### 2.6. Ethics Statement

This study was conducted in accordance with the Declaration of Helsinki and approved by the Peking University Institutional Review Board (protocol code IRB00001052-24163, 19 December 2024) and Ethics Committee of Shenzhen Center for Disease Control and Prevention (protocol code SZCDC-IRB2024076, 8 August 2024). Patient consent was waived due to the use of historical monitoring data.

## 3. Results

**Figure 1:**
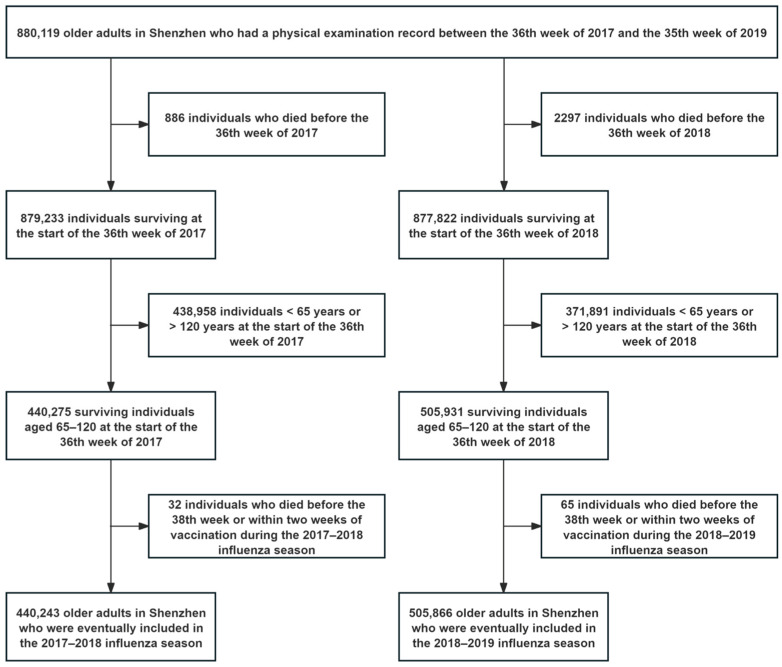
Inclusion process for research subjects during the 2017–2018 and 2018–2019 influenza seasons.

The initial number of older individuals in Shenzhen with a physical examination record was 880,119, and no subjects were missing information on date of death, underlying cause of death, or influenza vaccination date. After excluding individuals who had died before baseline, individuals younger than 65 years or older than 120 years, and individuals who died within two weeks of baseline initiation or within two weeks of vaccination, 440,243 subjects were included in the 2017–2018 influenza season and 505,866 in the 2018–2019 influenza season (Figure 1).

### 3.1. Propensity Score Matching

Before propensity score matching, during the 2017–2018 influenza season, the influenza vaccination rate was 6.53% and the all-cause mortality rate was 0.31%, of which 39.67% was CCVD mortality. During the 2018–2019 influenza season, the influenza vaccination rate was 4.02%, of which 62.56% was current-season-only vaccination, and the all-cause mortality rate was 0.41%, of which 39.85% was CCVD mortality. There were significant differences between the control and exposure groups at baseline in terms of demographic information, behavioral factors, and underlying conditions.

After propensity score nearest-neighbor matching, a total of 28,730 vaccinated and 114,920 unvaccinated individuals were matched at a 1:4 ratio during the 2017–2018 influenza season, and a total of 20,315 vaccinated and 81,260 unvaccinated individuals were matched during the 2018–2019 influenza season (Tables S1 and S2). Among those with vaccination records for the 2018–2019 influenza season, 5471 who were vaccinated for the current season only and 5471 who were vaccinated for two continuous seasons were matched 1:1, with all-cause mortality rates of 0.31% and 0.62%, respectively. We observed that in the vaccinated population, all deaths were retained after propensity score matching. Considering the potential for the algorithm to randomly select among candidates with identical propensity scores, we repeated the matching process using multiple random seed values to eliminate the possibility of chance-driven anomalies. The results consistently showed that all deceased individuals in the vaccinated population were retained. The mean age of the vaccinated subjects was 72.04 years, 53.48% were female, 57.56% of the subjects had medical insurance, 96.62% received vaccines from the immunization program, 83.95% received IIV3, and 78.12% received vaccines from the Changchun Institute of Biological Products. The control and exposure groups were comparable in terms of demographic information, behavioral factors, and underlying conditions (SMD < 0.10) (Table 1).

### 3.2. Survival Analysis

**Figure 2:**
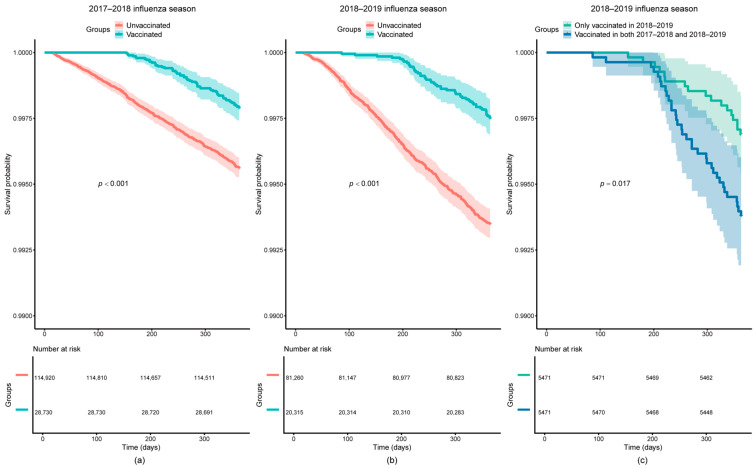
Kaplan–Meier curves of influenza vaccination and all-cause survival probability. (**a**) During the 2017–2018 influenza season, the all-cause survival probability was higher in the influenza-vaccinated group compared to the unvaccinated group (*p* < 0.001); (**b**) during the 2018–2019 influenza season, the all-cause survival probability was higher in the influenza-vaccinated group compared to the unvaccinated group (*p* < 0.001); (**c**) the all-cause survival probability was higher in those who were only vaccinated in 2018–2019 compared to those who were vaccinated in two continuous seasons in 2017–2018 and 2018–2019 (*p* = 0.017).

**Figure 3:**
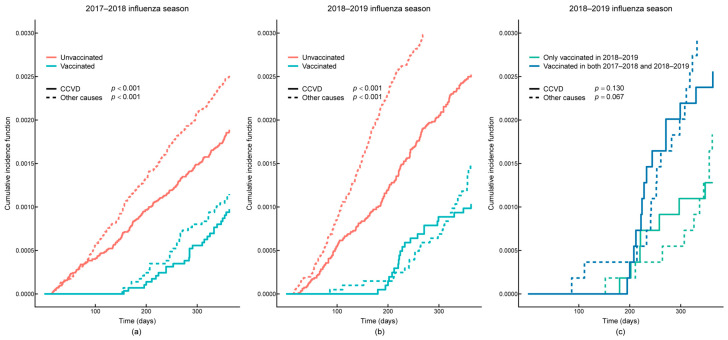
Cumulative incidence function curves for influenza vaccination and cardio-cerebral vascular disease (CCVD) mortality after accounting for competing risks of death from other causes. (**a**) During the 2017–2018 influenza season, the cumulative incidence of CCVD mortality was lower in the influenza-vaccinated group compared to the unvaccinated group after accounting for the competing risk of mortality from other causes (*p* < 0.001); (**b**) during the 2018–2019 influenza season, the cumulative incidence of CCVD mortality was lower in the influenza-vaccinated group compared to the unvaccinated group after accounting for the competing risk of mortality from other causes (*p* < 0.001); (**c**) after accounting for the competing risk of mortality from other causes, there was no significant difference in the cumulative incidence of CCVD mortality between those who were vaccinated in two continuous seasons in 2017–2018 and 2018–2019 and those who were vaccinated only in 2018–2019 (*p* = 0.130).

As shown in the Kaplan–Meier survival curves, all-cause survival probability was higher in the vaccinated group compared to the unvaccinated group during the 2017–2018 and 2018–2019 influenza seasons (*p* < 0.001). The all-cause survival probability was higher in the current-season-only vaccination group compared to the continuous two-season vaccination group (*p* = 0.017) (Figure 2). As can be seen from the cumulative incidence function curves, the influenza-vaccinated group had a lower cumulative incidence of CCVD mortality compared with the unvaccinated group in the 2017–2018 and 2018–2019 influenza seasons after accounting for the competing risks of mortality from other causes (*p* < 0.001). There was no significant difference in the cumulative incidence of CCVD mortality between the continuous two-season vaccination group and the current-season-only vaccination group (*p* = 0.130) (Figure 3).

In the Cox multivariable regression analysis, we included the interaction term between influenza vaccination and log survival time in the model. The regression coefficient for this interaction term was not statistically significant (*p* > 0.05), indicating that the proportional hazards assumption was met for the constructed model. After adjusting for demographic characteristics, behavioral factors, and underlying conditions, influenza vaccination during the 2017–2018 and 2018–2019 influenza seasons was associated with a 39% (HR = 0.61, 95% CI: 0.47–0.80) and 55% (HR = 0.45, 95% CI: 0.33–0.60) reduction in all-cause mortality risk, respectively, compared to no vaccination. In addition, vaccination only during the 2018–2019 season reduced the risk of all-cause mortality by 44% (HR = 0.56, 95% CI: 0.31–0.99) compared to continuous two-season vaccination. In the Fine–Gray multivariable competing risk models, the results for the 2018–2019 influenza season showed that influenza vaccination was associated with a 46% reduction in the risk of CCVD mortality after adjustment (HR = 0.54, 95% CI: 0.34–0.84) compared with no vaccination. No significant difference in the risk of CCVD mortality was observed between the current-season-only vaccination group and the continuous two-season vaccination group. There was no multicollinearity among the variables included in the multivariable analysis, as all generalized variance inflation factors were close to 1. After analyses using caliper matching, nested case–control design with conditional logistic regression, and Poisson’s regression, respectively, the results were all similar to those of the main analysis, suggesting that the findings were robust (Table 2).

Subgroup analyses revealed a stronger protective effect of influenza vaccination against all-cause mortality in individuals with medical insurance during both influenza seasons (*p* < 0.001). In the 2017–2018 season, this effect was more pronounced in those without diabetes (*p* = 0.045), while in the 2018–2019 season, it was stronger among females (*p* = 0.029) and individuals aged over 75 years (*p* = 0.008) (Table 3).

After stratifying CCVD into ischemic heart disease, cerebrovascular disease, and other circulatory system diseases, the analysis in the total population showed no significant association between influenza vaccination and reduced risk of mortality from ischemic heart disease, cerebrovascular disease, or other circulatory system disease during the 2017–2018 influenza season. However, during the 2018–2019 influenza season, influenza vaccination was associated with a 51% (HR = 0.49, 95% CI: 0.13–0.80) reduction in the risk of cerebrovascular disease mortality (Table 4).

## 4. Discussion

### 4.1. Interpretation of Results

This study found that older adults vaccinated only in the 2018–2019 influenza season had a lower risk of all-cause mortality (HR = 0.56, 95% CI: 0.31–0.99) compared with those vaccinated in both the 2017–2018 and 2018–2019 seasons, consistent with previous epidemiologic studies suggesting that repeated influenza vaccination may lead to decreased protection [12,13,30,31]. Song et al. found that the protective effect of influenza vaccines against influenza A virus infection differed significantly between continuous vaccination and current-season-only vaccination (ΔVE = 53%, 95% CI: 15–77%), with lower effectiveness observed in continuously vaccinated individuals [32]. Kwong et al. reported that among participants vaccinated in the current season but not in the previous 10 seasons, VE (VE = 34%, 95% CI: 9–52%) was higher compared to those vaccinated in 1–3 previous seasons (VE = 26%, 95% CI: 13–37%), 4–6 previous seasons (VE = 24%, 95% CI: 15–33%), 7–8 previous seasons (VE = 13%, 95% CI: 2–22%), or 9–10 previous seasons (VE = 7%, 95% CI: −4–16%), with a significant trend (*p* = 0.001) [30]. Saito et al. demonstrated that compared to individuals unvaccinated in the previous season (OR = 46%, 95% CI: 26–60%), those vaccinated in the previous season had a significantly reduced protective effect against influenza infection (OR = 19%, 95% CI: 0–35%) [33]. Immunological studies have also shown that the antibody response to influenza viruses may gradually diminish as the number of vaccinations increases [34,35]. Petrie et al. observed lower hemagglutination-inhibition antibody titers in individuals vaccinated for two continuous seasons compared to those vaccinated only in the current season [36]. Sherman et al. reported that individuals with fewer prior seasonal influenza vaccinations exhibited a stronger antibody-secreting cell response following vaccination [37]. Sullivan et al. found antibody titers were lowest in participants who had been vaccinated in the prior five years but highest among those with zero or one prior vaccination [38]. Sanyal et al. noted that repeated vaccination with IIV3 weakened B-cell responses [35]. The antigenic distance hypothesis, which considers the relationship between vaccine strain composition and circulating strains, has been proposed to explain these potential associations [39]. According to the WHO’s annual recommendations for influenza vaccine composition in the Northern Hemisphere, the 2018–2019 vaccine replaced the strains of A (H3N2) and B (Victoria) compared to the 2017–2018 vaccine, and the A (H1N1) and B (Yamagata) strains remained unchanged [40,41]. Surveillance data on influenza virus in Shenzhen indicated that the dominant strains during the 2017–2018 season were B (Yamagata) and A (H1N1), while in the 2018–2019 season, the dominant strains were A (H1N1) and B (Victoria) [42]. The potential mechanism underlying the results may be attributed to the similarity of vaccine strains across the two continuous influenza seasons, yet the circulating strains underwent changes. If individuals vaccinated in both seasons developed an immune response predominantly influenced by the first season’s vaccine strains, the protective effect of the second season’s vaccine might be reduced [39]. Given that the exact mechanisms are not yet fully understood, more randomized controlled trials are needed in the future to clarify the variation in the protective effect of influenza vaccination over seasons and to provide insight into the mechanisms of individual immune response.

Although this study found that current-season-only influenza vaccination was more effective than continuous two-season vaccination, this does not negate the necessity of annual vaccination or mean that the population should choose a non-continuous vaccination strategy. First, as noted by Lim et al. in their study investigating the impact of continuous influenza vaccination on medically attended acute respiratory illness [43], the finding that continuous influenza vaccination may reduce the protective effect is inconclusive due to the potential for residual confounding in the dataset. In addition, the results of this study reflect only the situation in Shenzhen during the 2017–2018 and 2018–2019 seasons and cannot be simply generalized to other time periods or regions. For instance, the National Advisory Committee on Immunization in Canada reported no significant difference or consistent trend in VE between continuous two-season vaccination and current-season-only vaccination [44]. Nichols found no significant reduction in VE against the influenza A (H3N2) strain in individuals vaccinated across two continuous seasons, and even higher VE against influenza B strains was observed [45]. Similarly, Kitamura reported no association between annual seasonal influenza vaccination and a decline in VE among older adults [46]. Cheng et al. found that VE was 51% (95% CI: 45–57%) in individuals vaccinated over two continuous seasons compared to 33% (95% CI: 17–47%) in those vaccinated only in the current season, with similar patterns observed for influenza A (H1N1), A (H3N2), and B strains [47]. Yang et al. reported no significant increase in influenza risk among individuals vaccinated across two continuous seasons compared to those vaccinated in the current season only (OR = 1.22, 95% CI: 0.94–1.58) [48]. Huang et al. observed no significant difference in serum antibody titer between groups receiving repeated IIV3 vaccination and those receiving their first vaccination [49]. Ye et al. found that continuous vaccination provided similar or enhanced protection compared to a single vaccination [50]. These findings suggest that many studies have not demonstrated a consistent association between continuous vaccination over two or more seasons and a reduction in VE, so no definitive conclusion can yet be drawn regarding whether continuous influenza vaccination leads to diminished protective effectiveness. Furthermore, our results highlighted that compared to the unvaccinated group, influenza vaccination during the 2017–2018 and 2018–2019 influenza seasons was associated with a significant reduction in all-cause mortality risk by 39% (HR = 0.61, 95% CI: 0.47–0.80) and 55% (HR = 0.45, 95% CI: 0.33–0.60), respectively. Previous meta-analyses have also consistently reported a 30–50% reduction in mortality risk associated with influenza vaccination [9,51,52]. This result reflects that, regardless of prior vaccination status, vaccination during the influenza season was associated with a significant reduction in the risk of mortality compared with unvaccinated individuals. Therefore, older adults should maintain vaccination coverage each influenza season for effective protection.

In line with previous studies evaluating the protective effects of influenza vaccination against cardiovascular events [53], the present study also found significant protection from influenza vaccination against CCVD mortality within the influenza season in older adults. Notably, a significant association between influenza vaccination and reduced risk of cerebrovascular disease mortality (HR = 0.49, 95% CI: 0.13–0.80) was observed during the 2018–2019 influenza season. This finding suggests that the protective effect of influenza vaccination on CCVD mortality in this study may primarily stem from its protection against cerebrovascular disease. Similarly, Modin et al. reported a significant association between influenza vaccination and reduced risk of stroke-related mortality [54,55], and Bacurau et al. demonstrated that influenza vaccination campaigns in Brazil effectively decreased mortality from cerebrovascular disease among older adults [56]. Studies by Zahhar et al. and Pang et al. found that influenza vaccination was associated with reduced mortality in stroke patients [57,58]. Possible mechanisms of influenza vaccine protection against CCVD mortality include reducing the onset of the inflammatory cascade by activating the immune system and preventing deaths from CCVD progression by facilitating smaller and more stable atherosclerotic plaques [59,60]. In addition, influenza vaccination could indirectly reduce infection-induced pro-inflammatory and pro-coagulant pathways as well as cardiovascular system stress responses by lessening the incidence of acute respiratory infections [61]. The findings suggested that the maintenance of cardiovascular system health during the influenza season may require the protective effects of influenza vaccination.

Furthermore, this study found that the vaccination rates among older adults in Shenzhen during the 2017–2018 and 2018–2019 influenza seasons were only 6.53% and 4.02%, respectively, slightly higher than the overall influenza vaccination rate of older adults in China [18] but still lower than that of developed countries and the WHO-recommended level of 75% [19,20,21]. Despite the implementation of a free influenza vaccination policy for older adults in Shenzhen, the actual coverage remains suboptimal. Thus, efforts to strengthen vaccination promotion and education among older adults should be prioritized to increase coverage and maximize the public health benefits of influenza vaccination in the population.

This study also found a stronger association between influenza vaccination and a lower risk of all-cause mortality in the insured group compared with the self-pay group. This may be due to the fact that the insured population has better access to health care services, including vaccination, and that the insured population may have higher health awareness with greater involvement in health promotion and disease prevention behaviors. Influenza vaccination interacted with other health-promoting behaviors, leading to stronger protective effects. However, the insured may represent a group of healthier or health-conscious individuals with a greater willingness to choose vaccination and participate in wellness programs. Given that this study was conducted on the physical examination database, possible selection bias could also have led to the appearance that influenza vaccine protection was stronger in the insured group.

### 4.2. Limitations of the Study

First, the observation period in this study was focused on influenza seasons of no more than one year, which may have limited the assessment of long-term vaccine effects. Second, this study focused on mortality events, an ultimate outcome that is influenced by numerous factors. Although we adjusted for known confounders in the multifactorial models, there were still many unmeasured potential confounders that could have affected the association between influenza vaccination and mortality, such as clinical indicators, chronic diseases, and previous influenza vaccination status. Due to the limitations of the dataset, we were unable to account for vaccination history from earlier influenza seasons, which may have had implications for the study results. Third, this study was conducted in a southern city in China, where influenza shows a bimodal pattern within a single season, and only two influenza seasons were included in the analysis. Therefore, caution should be exercised when extrapolating our conclusions, with consideration of the local epidemiological characteristics of influenza. Finally, this study was a retrospective cohort study, and the evidence derived was based on observation rather than strict randomization and intervention. Thus, although valuable information on the association between influenza vaccination and reduced risk of mortality could be provided, confirmation of causality requires further research and validation.

## 5. Conclusions

Influenza vaccination, regardless of prior vaccination status, was associated with reduced risk of all-cause and CCVD mortality in older adults, highlighting the importance of maintaining vaccination coverage during influenza seasons. Although this study found that the protective effect of current-season-only vaccination was greater than that of continuous two-season vaccination, this conclusion is not definitive. Further randomized controlled trials, as well as in-depth investigations into individual immune response mechanisms, are needed to clarify the changes in protection associated with repeated seasonal influenza vaccination.

## Figures and Tables

**Table 1:** Distribution of covariates among those vaccinated before and after propensity score matching during the 2018–2019 influenza season.

Variable	Group	Before Propensity Score Matching				After Propensity Score Matching			
		Overall	Vaccinated in Both 2017–2018 and 2018–2019	Only Vaccinated in 2018–2019	SMD	Overall	Vaccinated in Both 2017–2018 and 2018–2019	Only Vaccinated in 2018–2019	SMD
Sample size		20,315	12,710	7605		10,942	5471	5471	
Death, *n* (%)	No	20,264 (99.75)	12,676 (99.73)	7588 (99.78)	0.009	10,891 (99.53)	5437 (99.38)	5454 (99.69)	0.046
	Yes	51 (0.25)	34 (0.27)	17 (0.22)		51 (0.47)	34 (0.62)	17 (0.31)	
Gender, *n* (%)	Male	9555 (47.03)	6075 (47.80)	3480 (45.76)	0.041	5090 (46.52)	2571 (46.99)	2519 (46.04)	0.019
	Female	10,760 (52.97)	6635 (52.20)	4125 (54.24)		5852 (53.48)	2900 (53.01)	2952 (53.96)	
Age, mean (SD)		72.39 (5.82)	72.61 (5.83)	72.02 (5.77)	0.102	72.04 (5.68)	72.13 (5.68)	71.95 (5.68)	0.030
Ethnicity, *n* (%)	Han	20,264 (99.75)	12,680 (99.76)	7584 (99.72)	0.008	10,912 (99.73)	5455 (99.71)	5457 (99.74)	0.007
	Minority	51 (0.25)	30 (0.24)	21 (0.28)		30 (0.27)	16 (0.29)	14 (0.26)	
Marital status, *n* (%)	Unmarried	10 (0.05)	6 (0.05)	4 (0.05)	0.028	2 (0.02)	1 (0.02)	1 (0.02)	0.026
	Married	19,288 (94.94)	12,092 (95.14)	7196 (94.62)		10,353 (94.62)	5192 (94.90)	5161 (94.33)	
	Divorce	74 (0.36)	49 (0.39)	25 (0.33)		32 (0.29)	16 (0.29)	16 (0.29)	
	Widowhood	943 (4.64)	563 (4.43)	380 (5.00)		555 (5.07)	262 (4.79)	293 (5.36)	
Education level, *n* (%)	Junior high school and below	10,668 (52.51)	6368 (50.10)	4300 (56.54)	0.130	5730 (52.37)	2834 (51.80)	2896 (52.93)	0.024
	High school	5808 (28.59)	3845 (30.25)	1963 (25.81)		3070 (28.06)	1561 (28.53)	1509 (27.58)	
	University and above	3839 (18.90)	2497 (19.65)	1342 (17.65)		2142 (19.58)	1076 (19.67)	1066 (19.48)	
Insurance type, *n* (%)	Insurance payment	12,668 (62.36)	8847 (69.61)	3821 (50.24)	0.403	6298 (57.56)	3208 (58.64)	3090 (56.48)	0.044
	Self-funded payment	7647 (37.64)	3863 (30.39)	3784 (49.76)		4644 (42.44)	2263 (41.36)	2381 (43.52)	
Occupational type, *n* (%)	No occupation	7719 (38.00)	4527 (35.62)	3192 (41.97)	0.137	4286 (39.17)	2118 (38.71)	2168 (39.63)	0.019
	production technology	8839 (43.51)	5672 (44.63)	3167 (41.64)		4641 (42.41)	2337 (42.72)	2304 (42.11)	
	Management	3757 (18.49)	2511 (19.76)	1246 (16.38)		2015 (18.42)	1016 (18.57)	999 (18.26)	
Dietary habits, *n* (%)	Balanced	18,814 (92.61)	11,808 (92.90)	7006 (92.12)	0.031	10,111 (92.41)	5063 (92.54)	5048 (92.27)	0.021
	Mainly meat	272 (1.34)	166 (1.31)	106 (1.39)		166 (1.52)	81 (1.48)	85 (1.55)	
	Mainly vegetarian	1118 (5.50)	667 (5.25)	451 (5.93)		605 (5.53)	294 (5.37)	311 (5.68)	
	Salt, oil, and sugar addiction	111 (0.55)	69 (0.54)	42 (0.55)		60 (0.55)	33 (0.60)	27 (0.49)	
Frequency of exercise, *n* (%)	Never	3200 (15.75)	1931 (15.19)	1269 (16.69)	0.041	1816 (16.60)	907 (16.58)	909 (16.61)	0.028
	Occasionally	911 (4.48)	569 (4.48)	342 (4.50)		491 (4.49)	260 (4.75)	231 (4.22)	
	More than once a week	1556 (7.66)	977 (7.69)	579 (7.61)		841 (7.69)	427 (7.80)	414 (7.57)	
	Every day	14,648 (72.10)	9233 (72.64)	5415 (71.20)		7794 (71.23)	3877 (70.86)	3917 (71.60)	
Smoking, *n* (%)	Smoke	1632 (8.03)	987 (7.77)	645 (8.48)	0.038	850 (7.77)	421 (7.70)	429 (7.84)	0.007
	Quit smoking	2064 (10.16)	1334 (10.50)	730 (9.60)		1076 (9.83)	542 (9.91)	534 (9.76)	
	Never smoke	16,619 (81.81)	10,389 (81.74)	6230 (81.92)		9016 (82.40)	4508 (82.40)	4508 (82.40)	
Alcohol consumption, *n* (%)	Never	17,083 (84.09)	10,687 (84.08)	6396 (84.10)	0.027	9236 (84.41)	4624 (84.52)	4612 (84.30)	0.016
	Occasionally	1930 (9.50)	1233 (9.70)	697 (9.17)		955 (8.73)	482 (8.81)	473 (8.65)	
	Often or every day	1302 (6.41)	790 (6.22)	512 (6.73)		751 (6.86)	365 (6.67)	386 (7.06)	
BMI, *n* (%)	Normal	12,184 (59.98)	7566 (59.53)	4618 (60.72)	0.039	6671 (60.97)	3329 (60.85)	3342 (61.09)	0.023
	Thin	701 (3.45)	418 (3.29)	283 (3.72)		401 (3.66)	194 (3.55)	207 (3.78)	
	Overweight	6614 (32.56)	4213 (33.15)	2401 (31.57)		3429 (31.34)	1735 (31.71)	1694 (30.96)	
	Obesity	816 (4.02)	513 (4.04)	303 (3.98)		441 (4.03)	213 (3.89)	228 (4.17)	
HGB, *n* (%)	Non anemic	19,414 (95.56)	12,135 (95.48)	7279 (95.71)	0.012	10,461 (95.60)	5220 (95.41)	5241 (95.80)	0.019
	Anemia	901 (4.44)	575 (4.52)	326 (4.29)		481 (4.40)	251 (4.59)	230 (4.20)	
SCR, *n* (%)	Normal	19,261 (94.81)	12,039 (94.72)	7222 (94.96)	0.011	10,401 (95.06)	5196 (94.97)	5205 (95.14)	0.017
	Low	615 (3.03)	392 (3.08)	223 (2.93)		318 (2.91)	157 (2.87)	161 (2.94)	
	High	439 (2.16)	279 (2.20)	160 (2.10)		223 (2.04)	118 (2.16)	105 (1.92)	
Hypertension, *n* (%)	No	9952 (48.99)	6001 (47.21)	3951 (51.95)	0.095	5498 (50.25)	2711 (49.55)	2787 (50.94)	0.028
	Yes	10,363 (51.01)	6709 (52.79)	3654 (48.05)		5444 (49.75)	2760 (50.45)	2684 (49.06)	
Diabetes, *n* (%)	No	16,045 (78.98)	9899 (77.88)	6146 (80.82)	0.072	8742 (79.89)	4374 (79.95)	4368 (79.84)	0.003
	Yes	4270 (21.02)	2811 (22.12)	1459 (19.18)		2200 (20.11)	1097 (20.05)	1103 (20.16)	
Immunization program, *n* (%)	No	2424 (11.93)	180 (1.42)	2244 (29.51)	0.843	370 (3.38)	180 (3.29)	190 (3.47)	0.010
	Yes	17,891 (88.07)	12,530 (98.58)	5361 (70.49)		10,572 (96.62)	5291 (96.71)	5281 (96.53)	
Vaccine type, *n* (%)	IIV3	18,152 (89.35)	11,512 (90.57)	6640 (87.31)	0.105	9186 (83.95)	4598 (84.04)	4588 (83.86)	0.005
	IIV4	2163 (10.65)	1198 (9.43)	965 (12.69)		1756 (16.05)	873 (15.96)	883 (16.14)	
Vaccine manufacturer, *n* (%)	HUALAN BIO	2829 (13.93)	1625 (12.79)	1204 (15.83)	0.126	2095 (19.15)	1035 (18.92)	1060 (19.37)	0.048
	Changchun Institute of Biological Products	17,128 (84.31)	10,917 (85.89)	6211 (81.67)		8548 (78.12)	4307 (78.72)	4241 (77.52)	
	Sanofi Pasteur	358 (1.76)	168 (1.32)	190 (2.50)		299 (2.73)	129 (2.36)	170 (3.11)	

Abbreviations: SMD, standardized mean difference; SD, standard deviation; BMI, body mass index; HGB, hemoglobin; SCR, serum creatinine; IIV, inactivated influenza vaccine.

**Table 2:** Association of influenza vaccination with all-cause mortality and CCVD mortality using propensity score matching, nested case–control design, and Poisson’s regression.

Influenza Season and Exposure Group	Study Design or Analytical Approach	All-Cause Mortality		CCVD Mortality	
		HR (95% CI)	*p*-Value	HR (95% CI)	*p*-Value
2017–2018, vaccinated *	Main analysis	0.61 (0.47, 0.80)	<0.001	0.68 (0.46, 1.02)	0.060
	PSM with caliper values	0.61 (0.47, 0.80)	<0.001	0.68 (0.46, 1.01)	0.059
	Nested case–control design	0.63 (0.48, 0.83)	0.001	0.69 (0.46, 1.05)	0.087
	Poisson’s regression	0.61 (0.47, 0.80)	<0.001	0.68 (0.46, 1.02)	0.059
2018–2019, vaccinated *	Main analysis	0.45 (0.33, 0.60)	<0.001	0.54 (0.34, 0.84)	0.006
	PSM with caliper values	0.45 (0.33, 0.60)	<0.001	0.54 (0.34, 0.84)	0.006
	Nested case–control design	0.48 (0.36, 0.65)	<0.001	0.58 (0.37, 0.92)	0.020
	Poisson’s regression	0.45 (0.34, 0.60)	<0.001	0.53 (0.34, 0.84)	0.006
2018–2019, only vaccinated in 2018–2019 *,#	Main analysis	0.56 (0.31, 0.99)	0.049	0.54 (0.21, 1.39)	0.200
	PSM with caliper values	0.57 (0.31, 0.99)	0.049	0.54 (0.21, 1.38)	0.190
	Nested case–control design	0.43 (0.19, 0.96)	0.039	0.50 (0.13, 1.87)	0.299
	Poisson’s regression	0.55 (0.31, 1.00)	0.049	0.59 (0.24, 1.48)	0.261

* Models adjusted for gender, age, ethnicity, marital status, education level, insurance type, occupational type, dietary habits, frequency of exercise, smoking, alcohol consumption, BMI, HGB, SCR, hypertension, and diabetes. # Models adjusted for immunization program, vaccine type, and vaccine manufacturer. The HR derived from the main analysis and PSM with caliper values, the OR derived from the nested case–control design, and the RR derived from the Poisson regression are uniformly expressed as HR here. Abbreviations: HR, hazard ratio; CI, confidence interval; PSM, propensity score matching; OR, odds ratio; RR, relative risk.

**Table 3:** Subgroup analysis of the association between influenza vaccination and all-cause mortality in the total study population during the 2017–2018 and 2018–2019 influenza seasons.

Variable	Group	2017–2018 Influenza Season		2018–2019 Influenza Season	
		HR (95%CI)	Interaction *p*-Value	HR (95%CI)	Interaction *p*-Value
Gender	Female	0.43 (0.27, 0.68)	0.132	0.27 (0.16, 0.46)	0.029
	Male	0.62 (0.45, 0.87)		0.52 (0.36, 0.73)	
Age (years)	65–75	0.76 (0.48, 1.21)	0.092	0.70 (0.45, 1.11)	0.008
	>75	0.47 (0.34, 0.65)		0.31 (0.21, 0.46)	
Education level	Junior high school and below	0.51 (0.36, 0.71)	0.040	0.47 (0.33, 0.66)	0.224
	High school	0.40 (0.22, 0.72)		0.24 (0.12, 0.50)	
	University and above	1.26 (0.64, 2.50)		0.46 (0.21, 1.01)	
Insurance type	Insurance payment	0.34 (0.24, 0.50)	<0.001	0.28 (0.20, 0.41)	<0.001
	Self-funded payment	1.23 (0.81, 1.87)		1.16 (0.70, 1.93)	
Occupational type	Management	0.46 (0.25, 0.86)	0.826	0.45 (0.23, 0.86)	0.764
	Production technology	0.54 (0.30, 0.97)		0.45 (0.26, 0.77)	
	No occupation	0.57 (0.40, 0.80)		0.38 (0.24, 0.55)	
BMI	Normal	0.51 (0.36, 0.72)	0.646	0.36 (0.24, 0.54)	0.695
	Thin	0.64 (0.25, 1.65)		0.58 (0.17, 1.95)	
	Overweight	0.51 (0.30, 0.85)		0.43 (0.25, 0.73)	
	Obesity	0.91 (0.33, 2.47)		0.63 (0.24, 1.61)	
HGB	Non anemic	0.54 (0.40, 0.73)	0.639	0.43 (0.32, 0.59)	0.459
	Anemia	0.62 (0.36, 1.09)		0.35 (0.15, 0.80)	
Hypertension	No	0.59 (0.43, 0.82)	0.357	0.52 (0.35, 0.77)	0.237
	Yes	0.45 (0.28, 0.72)		0.31 (0.20, 0.49)	
Diabetes	No	0.49 (0.37, 0.66)	0.045	0.44 (0.33, 0.60)	0.249
	Yes	1.11 (0.56, 2.18)		0.21 (0.08, 0.58)	

**Table 4:** Association between influenza vaccination and mortality from ischemic heart disease, cerebrovascular disease, and other circulatory system diseases during the 2017–2018 and 2018–2019 influenza seasons in the total population.

Influenza Season	Cause of Death	Number of Events	ICD-10	HR (95% CI)	*p*-Value
2017–2018	CCVD	246	I00–I99	0.68 (0.46, 1.02)	0.060
	Ischemic heart disease	118	I20–I25	0.61 (0.33, 1.13)	0.120
	Cerebrovascular disease	82	I60–I69	0.65 (0.33, 1.29)	0.220
	Other circulatory system diseases	46	I00–I15, I26–I52, I70–I99	0.95 (0.43, 2.10)	0.890
2018–2019	CCVD	226	I00–I99	0.54 (0.34, 0.84)	0.006
	Ischemic heart disease	100	I20–I25	0.66 (0.36, 1.23)	0.190
	Cerebrovascular disease	82	I60–I69	0.49 (0.13, 0.80)	0.014
	Other circulatory system diseases	44	I00–I15, I26–I52, I70–I99	0.7 (0.26, 1.85)	0.470

Abbreviations: ICD-10, the International Statistical Classification of Diseases and Related Health Problems 10th Revision.

## Data Availability

The original data are not available to the public because they involve health information of all older adults in Shenzhen, and the researchers have signed a data confidentiality agreement. Further information about the research data can be requested from the corresponding authors.

## Supplementary Materials

The following supporting information can be downloaded at: https://www.mdpi.com/article/10.3390/vaccines13020164/s1, Table S1: Distribution of covariates across the total study population before and after propensity score matching during the 2017–2018 influenza season; Table S2: Distribution of covariates across the total study population before and after propensity score matching during the 2018–2019 influenza season.

## Abbreviations

The following abbreviations are used in this manuscript:

WHO	World Health Organization
VE	Vaccine effectiveness
OR	Odds ratio
BMI	Body mass index
HGB	Hemoglobin
SCR	Serum creatinine
ICD-10	The International Statistical Classification of Diseases and Related Health Problems 10th Revision
CCVD	Cardio-cerebral vascular diseases
SMD	Standardized mean difference
HR	Hazard ratio
CI	Confidence interval
