# Supplementary material for: Effect of Current-Season-Only Versus Continuous Two-Season Influenza Vaccination on Mortality in Older Adults: A Propensity-Score-Matched Retrospective Cohort Study
Source: Vaccines (Basel). 2025 Feb 8;13(2):164. doi: 10.3390/vaccines13020164 (PMC11860298; doi:10.3390/vaccines13020164)
Supplement: Supplementary file 1 [file vaccines-13-00164-s001.zip › vaccines-3438747-Supplementary Materials.pdf]

Table S1. Distribution of covariates across the total study population before and after propensity score matching during the 2017–2018 influenza season.

Variable	Group	Before propensity score matching				After propensity score matching			
		Overall	Unvaccinated	Vaccinated	SMD	Overall	Unvaccinated	Vaccinated	SMD
Sample size		440,243	411,513	28,730		143,650	114,920	28,730	
Death, *n* (%)	No	438,892 (99.69)	410,223 (99.69)	28,669 (99.79)	0.020	143,083 (99.61)	114,414 (99.56)	28,669 (99.79)	0.040
	Yes	1351 (0.31)	1290 (0.31)	61 (0.21)		567 (0.39)	506 (0.44)	61 (0.21)	
Gender, *n* (%)	Male	202,894 (46.09)	189,222 (45.98)	13,672 (47.59)	0.032	67,790 (47.19)	54,118 (47.09)	13,672 (47.59)	0.010
	Female	237,349 (53.91)	222,291 (54.02)	15,058 (52.41)		75,860 (52.81)	60,802 (52.91)	15,058 (52.41)	
Age, mean (SD)		71.36 (5.69)	71.30 (5.68)	72.26 (5.75)	0.167	72.28 (6.06)	72.28 (6.14)	72.26 (5.75)	0.004
Ethnicity, *n* (%)	Han	438,307 (99.56)	409,645 (99.55)	28,662 (99.76)	0.037	143,311 (99.76)	114,649 (99.76)	28,662 (99.76)	<0.001
	Minority	1936 (0.44)	1868 (0.45)	68 (0.24)		339 (0.24)	271 (0.24)	68 (0.24)	
Marital status, *n* (%)	Unmarried	799 (0.18)	769 (0.19)	30 (0.10)	0.030	152 (0.11)	122 (0.11)	30 (0.10)	0.006
	Married	413,941 (94.03)	386,803 (94.00)	27,138 (94.46)		135,583 (94.38)	108,445 (94.37)	27,138 (94.46)	
	Divorce	1640 (0.37)	1519 (0.37)	121 (0.42)		650 (0.45)	529 (0.46)	121 (0.42)	
	Widowhood	23,863 (5.42)	22,422 (5.45)	1441 (5.02)		7265 (5.06)	5824 (5.07)	1441 (5.02)	
Education level, *n* (%)	Junior high school and below	311,683 (70.80)	296,113 (71.96)	15,570 (54.19)	0.376	78,311 (54.52)	62,741 (54.60)	15,570 (54.19)	0.008
	High school	80,958 (18.39)	72,944 (17.73)	8014 (27.89)		39,830 (27.73)	31,816 (27.69)	8014 (27.89)	
	University and above	47,602 (10.81)	42,456 (10.32)	5146 (17.91)		25,509 (17.76)	20,363 (17.72)	5146 (17.91)	
Insurance type, *n* (%)	Insurance payment	130,835 (29.72)	113,593 (27.60)	17,242 (60.01)	0.691	86,657 (60.33)	69,415 (60.40)	17,242 (60.01)	0.008
	Self-funded payment	309,408 (70.28)	297,920 (72.40)	11,488 (39.99)		56,993 (39.67)	45,505 (39.60)	11,488 (39.99)	
Occupational type, *n* (%)	No occupation	261,462 (59.39)	249,744 (60.69)	11,718 (40.79)	0.418	58,771 (40.91)	47,053 (40.94)	11,718 (40.79)	0.011
	Production technology	135,592 (30.80)	123,654 (30.05)	11,938 (41.55)		59,970 (41.75)	48,032 (41.80)	11,938 (41.55)	

Variable	Group	Before propensity score matching				After propensity score matching			
		Overall	Unvaccinated	Vaccinated	SMD	Overall	Unvaccinated	Vaccinated	SMD
	Management	43,189 (9.81)	38,115 (9.26)	5074 (17.66)		24,909 (17.34)	19,835 (17.26)	5074 (17.66)	
Dietary habits, *n* (%)	Balanced	408,068 (92.69)	381,483 (92.70)	26,585 (92.53)	0.009	132,999 (92.59)	106,414 (92.60)	26,585 (92.53)	0.007
	Mainly meat	6306 (1.43)	5885 (1.43)	421 (1.47)		2066 (1.44)	1645 (1.43)	421 (1.47)	
	Mainly vegetarian	22,658 (5.15)	21,135 (5.14)	1523 (5.30)		7526 (5.24)	6003 (5.22)	1523 (5.30)	
	Salt, oil, and sugar addiction	3211 (0.73)	3010 (0.73)	201 (0.70)		1059 (0.74)	858 (0.75)	201 (0.70)	
Frequency of exercise, *n* (%)	Never	80,450 (18.27)	75,943 (18.45)	4507 (15.69)	0.075	22,623 (15.75)	18,116 (15.76)	4507 (15.69)	0.007
	Occasionally	21,694 (4.93)	20,300 (4.93)	1394 (4.85)		7086 (4.93)	5692 (4.95)	1394 (4.85)	
	More than once a week	35,592 (8.08)	33,226 (8.07)	2366 (8.24)		11,678 (8.13)	9312 (8.10)	2366 (8.24)	
	Every day	302,507 (68.71)	282,044 (68.54)	20,463 (71.23)		102,263 (71.19)	81,800 (71.18)	20,463 (71.23)	
Smoking, *n* (%)	Smoke	43,038 (9.78)	40,799 (9.91)	2239 (7.79)	0.076	11,117 (7.74)	8878 (7.73)	2239 (7.79)	0.008
	Quit smoking	44,830 (10.18)	41,932 (10.19)	2898 (10.09)		14,252 (9.92)	11,354 (9.88)	2898 (10.09)	
	Never smoke	352,375 (80.04)	328,782 (79.90)	23,593 (82.12)		118,281 (82.34)	94,688 (82.39)	23,593 (82.12)	
Alcohol consumption, *n* (%)	Never	369,956 (84.03)	345,732 (84.01)	24,224 (84.32)	0.037	121,491 (84.57)	97,267 (84.64)	24,224 (84.32)	0.009
	Occasionally	39,891 (9.06)	37,148 (9.03)	2743 (9.55)		13,467 (9.37)	10,724 (9.33)	2743 (9.55)	
	Often or every day	30,396 (6.90)	28,633 (6.96)	1763 (6.14)		8692 (6.05)	6929 (6.03)	1763 (6.14)	
BMI, *n* (%)	Normal	214,034 (48.62)	200,341 (48.68)	13,693 (47.66)	0.034	68,766 (47.87)	55,073 (47.92)	13,693 (47.66)	0.005
	Thin	16,911 (3.84)	15,902 (3.86)	1009 (3.51)		5007 (3.49)	3998 (3.48)	1009 (3.51)	
	Overweight	164,282 (37.32)	153,387 (37.27)	10,895 (37.92)		54,242 (37.76)	43,347 (37.72)	10,895 (37.92)	
	Obesity	45,016 (10.23)	41,883 (10.18)	3133 (10.90)		15,635 (10.88)	12,502 (10.88)	3133 (10.90)	
HGB, *n* (%)	Non anemic	410,193 (93.17)	383,427 (93.17)	26,766 (93.16)	<0.001	133,765 (93.12)	106,999 (93.11)	26,766 (93.16)	0.002

Variable	Group	Before propensity score matching				After propensity score matching			
		Overall	Unvaccinated	Vaccinated	SMD	Overall	Unvaccinated	Vaccinated	SMD
	Anemia	30,050 (6.83)	28,086 (6.83)	1964 (6.84)		9885 (6.88)	7921 (6.89)	1964 (6.84)	
SCR, *n* (%)	Normal	347,553 (78.95)	325,108 (79.00)	22,445 (78.12)	0.022	112,552 (78.35)	90,107 (78.41)	22,445 (78.12)	0.007
	Low	28,122 (6.39)	26,184 (6.36)	1938 (6.75)		9540 (6.64)	7602 (6.62)	1938 (6.75)	
	High	64,568 (14.67)	60,221 (14.63)	4347 (15.13)		21,558 (15.01)	17,211 (14.98)	4347 (15.13)	
Hypertension, *n* (%)	No	263,608 (59.88)	249,264 (60.57)	14,344 (49.93)	0.215	72,617 (50.55)	58,273 (50.71)	14,344 (49.93)	0.016
	Yes	176,635 (40.12)	162,249 (39.43)	14,386 (50.07)		71,033 (49.45)	56,647 (49.29)	14,386 (50.07)	
Diabetes, *n* (%)	No	372,906 (84.70)	350,072 (85.07)	22,834 (79.48)	0.147	114,878 (79.97)	92,044 (80.09)	22,834 (79.48)	0.015
	Yes	67,337 (15.30)	61,441 (14.93)	5896 (20.52)		28,772 (20.03)	22,876 (19.91)	5896 (20.52)	

Abbreviations: SMD, Standardized mean difference; SD, standard deviation; BMI, body mass index; HGB, hemoglobin; SCR, serum creatinine.

Table S2. Distribution of covariates across the total study population before and after propensity score matching during the 2018–2019 influenza season.

Variable	Group	Before propensity score matching				After propensity score matching			
		Overall	Unvaccinated	Vaccinated	SMD	Overall	Unvaccinated	Vaccinated	SMD
Sample size		505,866	485,551	20,315		101,575	81,260	20,315	
Death, *n* (%)	No	503,811 (99.59)	483,547 (99.59)	20,264 (99.75)	0.028	100,994 (99.43)	80,730 (99.35)	20,264 (99.75)	0.060
	Yes	2055 (0.41)	2004 (0.41)	51 (0.25)		581 (0.57)	530 (0.65)	51 (0.25)	
Gender, *n* (%)	Male	231,730 (45.81)	222,175 (45.76)	9555 (47.03)	0.026	47,694 (46.95)	38,139 (46.93)	9555 (47.03)	0.002
	Female	274,136 (54.19)	263,376 (54.24)	10,760 (52.97)		53,881 (53.05)	43,121 (53.07)	10,760 (52.97)	
Age, mean (SD)		71.43 (5.76)	71.39 (5.75)	72.39 (5.82)	0.173	72.42 (6.15)	72.43 (6.23)	72.39 (5.82)	0.006
Ethnicity, *n* (%)	Han	503,514 (99.54)	483,250 (99.53)	20,264 (99.75)	0.037	101,318 (99.75)	81,054 (99.75)	20,264 (99.75)	<0.001
	Minority	2352 (0.46)	2301 (0.47)	51 (0.25)		257 (0.25)	206 (0.25)	51 (0.25)	
Marital status, *n* (%)	Unmarried	865 (0.17)	855 (0.18)	10 (0.05)	0.042	43 (0.04)	33 (0.04)	10 (0.05)	0.008
	Married	477,956 (94.48)	458,668 (94.46)	19,288 (94.94)		96,343 (94.85)	77,055 (94.83)	19,288 (94.94)	
	Divorce	1714 (0.34)	1640 (0.34)	74 (0.36)		396 (0.39)	322 (0.40)	74 (0.36)	
	Widowhood	25,331 (5.01)	24,388 (5.02)	943 (4.64)		4793 (4.72)	3850 (4.74)	943 (4.64)	
Education level, *n* (%)	Junior high school and below	359,763 (71.12)	349,095 (71.90)	10,668 (52.51)	0.411	53,973 (53.14)	43,305 (53.29)	10,668 (52.51)	0.016
	High school	93,012 (18.39)	87,204 (17.96)	5808 (28.59)		28,746 (28.30)	22,938 (28.23)	5808 (28.59)	
	University and above	53,091 (10.50)	49,252 (10.14)	3839 (18.90)		18,856 (18.56)	15,017 (18.48)	3839 (18.90)	
Insurance type, *n* (%)	Insurance payment	145,340 (28.73)	132,672 (27.32)	12,668 (62.36)	0.753	63,900 (62.91)	51,232 (63.05)	12,668 (62.36)	0.014
	Self-funded payment	360,526 (71.27)	352,879 (72.68)	7647 (37.64)		37,675 (37.09)	30,028 (36.95)	7647 (37.64)	
Occupational type, *n* (%)	No occupation	301,966 (59.69)	294,247 (60.60)	7719 (38.00)	0.476	38,971 (38.37)	31,252 (38.46)	7719 (38.00)	0.017
	Production technology	155,662 (30.77)	146,823 (30.24)	8839 (43.51)		44,340 (43.65)	35,501 (43.69)	8839 (43.51)	
	Management	48,238 (9.54)	44,481 (9.16)	3757 (18.49)		18,264 (17.98)	14,507 (17.85)	3757 (18.49)	
Dietary habits, *n* (%)	Balanced	468,601 (92.63)	449,787 (92.63)	18,814 (92.61)	0.025	94,240 (92.78)	75,426 (92.82)	18,814 (92.61)	0.010
	Mainly meat	7203 (1.42)	6931 (1.43)	272 (1.34)		1349 (1.33)	1077 (1.33)	272 (1.34)	
	Mainly vegetarian	26,486 (5.24)	25,368 (5.22)	1118 (5.50)		5410 (5.33)	4292 (5.28)	1118 (5.50)	

Variable	Group	Before propensity score matching				After propensity score matching			
		Overall	Unvaccinated	Vaccinated	SMD	Overall	Unvaccinated	Vaccinated	SMD
	Salt, oil, and sugar addiction	3576 (0.71)	3465 (0.71)	111 (0.55)		576 (0.57)	465 (0.57)	111 (0.55)	
Frequency of exercise, *n* (%)	Never	92,215 (18.23)	89,015 (18.33)	3200 (15.75)	0.077	16,016 (15.77)	12,816 (15.77)	3200 (15.75)	0.003
	Occasionally	24,499 (4.84)	23,588 (4.86)	911 (4.48)		4582 (4.51)	3671 (4.52)	911 (4.48)	
	More than once a week	40,553 (8.02)	38,997 (8.03)	1556 (7.66)		7737 (7.62)	6181 (7.61)	1556 (7.66)	
	Every day	348,599 (68.91)	333,951 (68.78)	14,648 (72.10)		73,240 (72.10)	58,592 (72.10)	14,648 (72.10)	
Smoking, *n* (%)	Smoke	50,340 (9.95)	48,708 (10.03)	1632 (8.03)	0.071	8055 (7.93)	6423 (7.90)	1632 (8.03)	0.005
	Quit smoking	52,418 (10.36)	50,354 (10.37)	2064 (10.16)		10,272 (10.11)	8208 (10.10)	2064 (10.16)	
	Never smoke	403,108 (79.69)	386,489 (79.60)	16,619 (81.81)		83,248 (81.96)	66,629 (81.99)	16,619 (81.81)	
Alcohol consumption, *n* (%)	Never	422,991 (83.62)	405,908 (83.60)	17,083 (84.09)	0.031	85,619 (84.29)	68,536 (84.34)	17,083 (84.09)	0.008
	Occasionally	46,804 (9.25)	44,874 (9.24)	1930 (9.50)		9593 (9.44)	7663 (9.43)	1930 (9.50)	
	Often or every day	36,071 (7.13)	34,769 (7.16)	1302 (6.41)		6363 (6.26)	5061 (6.23)	1302 (6.41)	
BMI, *n* (%)	Normal	244,267 (48.29)	234,675 (48.33)	9592 (47.22)	0.031	48,137 (47.39)	38,545 (47.43)	9592 (47.22)	0.005
	Thin	18,968 (3.75)	18,269 (3.76)	699 (3.44)		3521 (3.47)	2822 (3.47)	699 (3.44)	
	Overweight	190,420 (37.64)	182,564 (37.60)	7856 (38.67)		39,123 (38.52)	31,267 (38.48)	7856 (38.67)	
	Obesity	52,211 (10.32)	50,043 (10.31)	2168 (10.67)		10,794 (10.63)	8626 (10.62)	2168 (10.67)	
HGB, *n* (%)	Non anemic	472,790 (93.46)	453,753 (93.45)	19,037 (93.71)	0.011	95,152 (93.68)	76,115 (93.67)	19,037 (93.71)	0.002
	Anemia	33,076 (6.54)	31,798 (6.55)	1278 (6.29)		6423 (6.32)	5145 (6.33)	1278 (6.29)	
SCR, *n* (%)	Normal	401,026 (79.28)	385,138 (79.32)	15,888 (78.21)	0.031	79,757 (78.52)	63,869 (78.60)	15,888 (78.21)	0.010
	Low	31,693 (6.27)	30,288 (6.24)	1405 (6.92)		6861 (6.75)	5456 (6.71)	1405 (6.92)	
	High	73,147 (14.46)	70,125 (14.44)	3022 (14.88)		14,957 (14.73)	11,935 (14.69)	3022 (14.88)	
Hypertension, *n* (%)	No	305,548 (60.40)	295,596 (60.88)	9952 (48.99)	0.241	50,468 (49.69)	40,516 (49.86)	9952 (48.99)	0.017
	Yes	200,318 (39.60)	189,955 (39.12)	10,363 (51.01)		51,107 (50.31)	40,744 (50.14)	10,363 (51.01)	
Diabetes, *n* (%)	No	428,960 (84.80)	412,915 (85.04)	16,045 (78.98)	0.158	80,824 (79.57)	64,779 (79.72)	16,045 (78.98)	0.018

Variable	Group	Before propensity score matching				After propensity score matching			
		Overall	Unvaccinated	Vaccinated	SMD	Overall	Unvaccinated	Vaccinated	SMD
	Yes	76,906 (15.20)	72,636 (14.96)	4270 (21.02)		20,751 (20.43)	16,481 (20.28)	4270 (21.02)	
